# Comparison on thermal transport properties of graphene and phosphorene nanoribbons

**DOI:** 10.1038/srep16215

**Published:** 2015-11-18

**Authors:** Xiao-Fang Peng, Ke-Qiu Chen

**Affiliations:** 1 Institute of Mathematics and Physics, Central South University of Forestry and Technology, Changsha 410004, China; 2 Department of Applied Physics, School of Physics and Electronics, Hunan University, Changsha 410082, China

## Abstract

We investigate ballistic thermal transport at low temperatures in graphene and phosphorene nanoribbons (PNRS) modulated with a double-cavity quantum structure. A comparative analysis for thermal transport in these two kinds of nanomaterials is made. The results show that the thermal conductance in PNRS is greater than that in graphene nanoribbons (GNRS). The ratio *k*_G_/*k*_P_ (*k*_G_ is the thermal conductivity in GNRS and *k*_P_ is the thermal conductivity in PNRS) decreases with lower temperature or for narrower nanoribbons, and increases with higher temperature or for wider nanoribbons. The greater thermal conductance and thermal conductivity in PNRS originate from the lower cutoff frequencies of the acoustic modes.

Two-dimensional graphene, a monolayer of carbon atoms arranged in a regular hexagonal lattice, has attracted considerable attention owing to its extraordinary mechanical, physical, and chemical properties^1^,^2^,^3^,^4^,^5^,^6^,^7^,^8^,^9^,^10^,^11^,^12^,^13^. Especially, graphene possesses extremely high thermal conductivity^14^ owing to the strong bonding of the light carbon atoms, which is promising to solve the problem of the lack of heat dissipation in ever-smaller integrated circuits with higher power densities. Recently, similar to graphene, phosphorene, in which each phosphorene atom is covalently connected to three neighboring phosphorene atoms, has also attracted a lot of research attention owing to its unique properties, such as extraordinary electronic^16^, optoelectronic^15^, and thermal transport properties^17^. Recently, quasi-one-dimensional graphene nanoribbons (GNRS) and phosphorene nanoribbons (PNRS) with various geometries have been designed^5^,^6^,^18^,^19^,^20^. Further studies have shown that the thermal transport in these quasi-one-dimensional sub-10-nm nanostructures is dominated by thermal phonons^15^,^21^. Many interesting thermal transport properties are found in these geometries, and the transmission of the phonons, or lattice vibrations, depends on shapes^6^, structural defects^22^,^23^, boundary conditions at ribbon edges^24^, strain^25^, nanoribbon width^20^, contact^26^, and so on. GNRS and PNRS can both be classified as zigzag (ZGNRS and ZPNRS for zigzag graphene nanoribbons and zigzag phosphorene nanoribbons) and armchair (AGNRS and APNRS for armchair graphene nanoribbons and armchair phosphorene nanoribbons) depending on their edge geometry. ZGNRS are metallic and AGNRS are metallic or semiconducting depending on the ribbon width. However, unlike GNRS, ZPNRS and APNRS are both semiconducting with a band gap of about 2 eV^27^. Interestingly, because these two types of materials have similar nanostructures and the thermal transport properties both sensitively depend on their geometrical structure and edge shape, it is natural to consider whether the thermal transport properties are also the same in such nanostructures.

It is known that the continuum model for elastic waves is an ideal method for simulating thermal transport properties at low temperatures, not only for micro- and nanostructures^28^ but also for few-atom width quantum structures^29^,^30^. The validity of this model of elasticity has also been discussed in detail by Wang *et al.*^31^. Many significant previous studies have reported the use of this model, such as the nonlinear thermal properties of three-terminal mesoscopic dielectric systems^28^, phonon-cavity-enhanced low-temperature thermal conductance of a semiconductor nanowire^32^, effect of defects on the thermal conductivity in a nanowire^33^, and confined phonon dispersion and group velocity for GNRS^34^, and so on. Herein, a comparative analysis for the thermal transport properties in GNRS and PNRS is made using this model. For the structures considered here, there exist three types of acoustic modes: namely, horizontally polarized shear SH mode with the polarization direction along the vertical direction of the plane, vertically polarized SV mode with the polarization direction along the vertical direction of the wave in the plane, and longitudinal P mode with the polarization direction along the propagation direction of the wave in the plane^32^. Because GNRS and PNRS are very thin, and this dimension is substantially smaller than the other two dimensions and also smaller than the wavelength of the elastic waves, the dynamics in the vertical direction of the plane can be neglected and the SH mode is decoupled from the SV and P modes^35^. It is also shown that the influence of the Hamiltonian mixing between SV and P on the thermal conductance is very small at low temperatures^36^, and these three modes have similar thermal transport properties. Therefore, we only focus on the thermal transport properties of the SH mode in these two kinds of nanomaterials. Our results show that despite the same chains across the GNRS and PNRS or the same lateral widths, the quantized thermal conductance plateau is wider and the low-temperature thermal conductance is less in GNRS than in PNRS. The ratio *k*_G_/*k*_P_ decreases with lower temperature or for narrower nanoribbons and increases with higher temperature or for wider nanoribbons. Additionally, *k*_P_ is greater than *k*_G_ in a certain low temperature range.

## Model and Method

**Figure 1:**
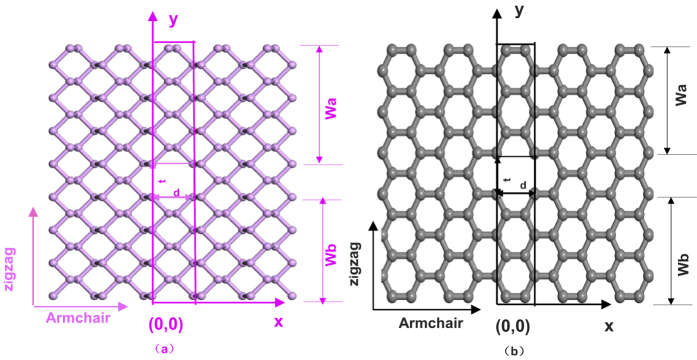
Lattice structures of (**a**) PNRS and (**b**) GNRS.

We model the PNRS and GNRS with cavities as illustrated in Fig. 1 (a,b). For thermal transport calculations in Fig. 1, we assume that the thermal current is along the armchair nanoribbons from the left to right or along the zigzag nanoribbons from the bottom to top. The nanoribbons are divided into three regions: the left semi-infinite nanoribbon region along the armchair direction or bottom semi-infinite nanoribbon region along the zigzag direction with temperature *T*_1_, the central scattering region with the double cavities, and the right semi-infinite nanoribbon region along the armchair direction or top semi-infinite nanoribbon region along the zigzag direction with temperature *T*_2_. Here, we assume that the Δ*T* (Δ*T* = *T*_1_ – *T*_2_, > 0) is so small that we can adopt the mean temperature *T* (*T *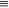 (*T*_1_ + *T*_2_)/2) as the temperature of the whole nanoribbon region. For the structures considered here, the expression of the thermal conductance can be written as:




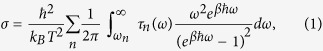




where *ω*_n_ is the cutoff frequency of the mode *n*, 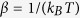, *k*_*B*_ is the Boltzmann’s constant, and 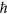 is the reduced Planck’s constant. 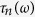 is the transmission rate of mode *n* for the left or bottom lead at frequency *ω* across the scattering region into the top or right lead. In the elastic approximation, the elastic equation of motion for the SH wave is:




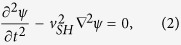




where 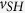 is the sound velocity of the SH mode. The solution to Eq. (2) along the armchair direction has a similar expression as that along the zigzag direction. Therefore, in the following discussion, we only describe the armchair-direction expression. The solution to Eq. (2) in the left region along the armchair direction can be written as:




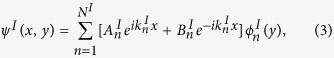




where 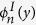 is the transverse wave function of acoustic mode *n* in the left region.

Using the stress-free boundary condition 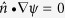 at the edges, the transverse wave function 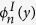 of acoustic mode *n* in the left region can be written as:




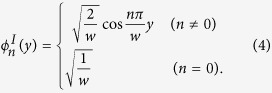




Note that the stress-free boundary condition allows the propagation of the zero acoustic mode, which is very important for predicting the quantum thermal conductance. By the energy conservation, 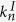 can be written as:




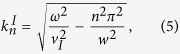




where ***ω*** is the incident phonon frequency. In the scattering region, the transverse wave function 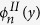 of acoustic mode *n* can be written as:




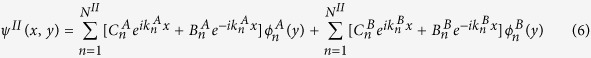




Using the stress-free boundary condition at the interfaces between the upper region of the cavity and the cavity region, and also between the lower region of the cavity and the cavity region, the transverse wave functions 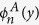 in the upper region of the cavity (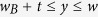), and 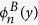 in the lower region of the cavity (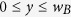) can be expressed as:




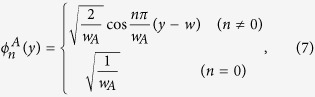




and




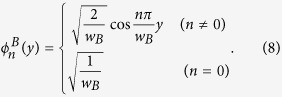




Here, 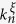 can be written as:




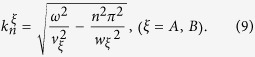




In the right region, the transverse wave function 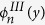 of acoustic mode *n* can be written as:




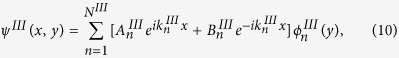




the transverse wave function 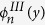 of acoustic mode *n* in the right region can be written as:




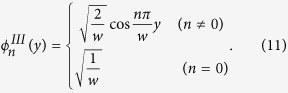




The sum over *n* includes all propagating and evanescent modes. However, in the real calculations, we consider all propagating modes and several of the lowest evanescent modes in our calculation, which can meet the desired precision. By considering the displacement and strain to be continuous at each interface and using the scattering matrix method, we can calculate the transmission co-efficient 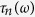, which is the key issue to predict the thermal conductance. In the calculations, we will employ the values of the constants of phosphorene and graphene as: sound velocity 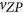 = 3.95 km/s for the zigzag direction and 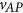 = 3.61 km/s for the armchair direction^37^. The thickness *D*_P_ = 0.5239 nm^24^ for phosphorene. The sound velocity 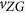 = 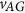 = 13.6 km/s^38^, and the thickness *D*_G_ = 0.335 nm^17^ for graphene.

## Results and Discussion

**Figure 2:**
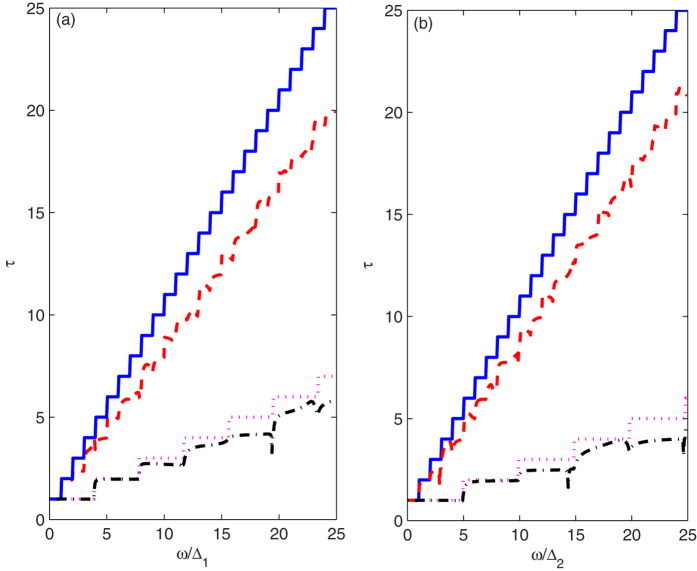
(**a,b**) correspond to the dependence of the total transmission probability on the reduced frequency ω/∆_1_ with ∆_1_ = πv_ZP_/w_1_ along the zigzag direction and ω/∆_2_ with ∆_2_ = πv_AP_/w_2_ along the armchair direction.The solid and dashed curves describe the transmission spectra of the PNRS with ideal structure and with double-cavity structure. The dotted and dash-dotted curves describe the transmission spectra of the GNRS with ideal structure and with double-cavity structure. The parameters are taken as the defect with the width t = 4.6 Å for ZPNRS, 3.3 Å for APNRS, 2.9 Å for ZGNRS, and 2.5 Å for AGNRS, and the length d = 3.3 Å for ZPNRS, 4.6 Å for APNRS, 2.5 Å for ZGNRS, and 2.9 Å for AGNRS. Here, w = Wa + Wb + t, and the lengths between the defect region and the two lateral sides of main quantum wire are Wa = Wb = 9.2 Å for ZPNRS, 8.3 Å for APNRS, 8.7 Å for ZGNRS, and 6.3 Å for AGNRS.

Figure 2(a,b) show the dependence of the total transmission coefficients on the reduced frequency ω/∆_1_ with ∆_1_ = πv_ZP_/w_1_, corresponding to the phonon transportation along the zigzag nanoribbon direction, and on the reduced frequency ω/∆_2_ with ∆_2_ = πv_AP_/w_2_, corresponding to the phonon transportation along the armchair nanoribbon direction. The solid and dashed curves describe the transmission spectra of the PNRS with ideal structure and with double-cavity structure. As a comparison, the dotted and dash-dotted curves represent the transmission spectra of the GNRS with ideal structure and with double-cavity structure, respectively. From the solid and dotted curves, we can clearly see that for a perfect nanoribbon, the phonon transmission curves exhibit quantization stepwise structures, and an abrupt jump is always located at an integer-reduced frequency for PNRS, and a non-integer reduced frequency for GNRS, where a new mode starts to be excited. The abrupt jump position of mode *n* occurs at the frequency ∆_ZG_ = nπv_ZG_/w_ZG_ with width w_ZG_ = 0.145(2 + 3(*k* − 2)/2) nm for ZGNRS, at the frequency ∆_ZP_ = nπv_ZP_/w_ZP_ with width w_ZP_ = 0.23*k* nm for ZPNRS, at the frequency ∆_AG_ = nπv_AG_/w_AG_ with width w_AG_ = 0.2511(*k* − 1) nm for AGNRS, and at the frequency ∆_AP_ = nπv_AP_/w_AP_ with width w_AP_ = 0.33(*k* − 1) nm for APNRS. Here, *k* is the chains (dimer lines) across the ribbon width following the conventional notation. Clearly, ∆_ZG_/∆_ZP_ = (v_ZG_w_ZP_)/(v_ZP_w_ZG_) = 3.9 for *k* = 10 and ∆_AG_/∆_AP_ = (v_AG_w_AP_)/(v_AP_w_AG_) = 4.95 for *k* = 7. This shows that the cutoff frequency of the mode *n* is far lower than that of the GNRS and the mode *n* in PNRS is excited easier. Therefore, the transmission rates of PNRS are obviously higher than those of GNRS, which means that the PNRS is more favorable for the acoustic phonon transport at the low frequency range. It can be found that in a quantum wire with a double-cavity scattering structure, the quantization steps are broken and the transmission spectra display complex peak-dip structures owing to the scattering of the double cavities. Clearly, comparing the transmission curves obtained from perfect nanoribbon samples in the higher-frequency region, the transmission curve of PNRS with a double-cavity scattering structure descends more obviously than that of GNRS with a double-cavity scattering structure. This is because at the higher-frequency region, more high-frequency phonon modes are excited in PNRS than that in GNRS and these phonon modes are scattered easily by the double-cavity scattering structure.

**Figure 3:**
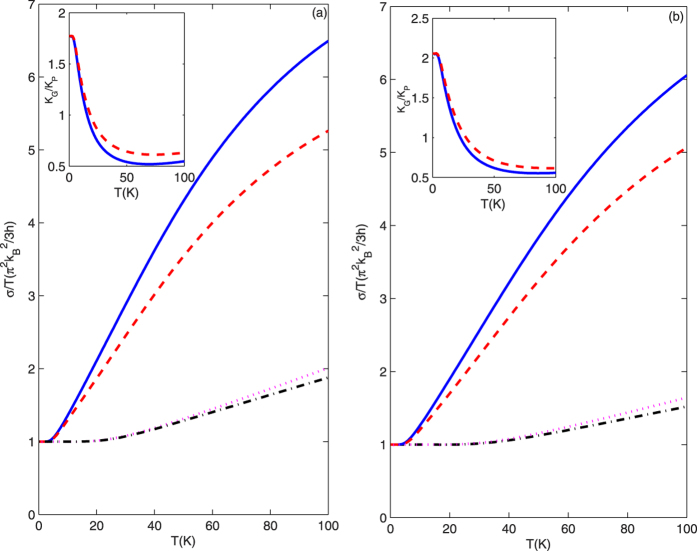
(**a,b**) correspond to the total reduced thermal conductance divided by temperature K/T reduced by the zero-temperature universal value π^2^k_B_^2^/3h as a function of temperature along the zigzag and along the armchair directions, respectively.The solid and dashed curves describe the total reduced thermal conductance of the PNRS with ideal structure and with double-cavity structure, respectively. The dotted and dash-dotted curves describe the total reduced thermal conductance of the GNRS with ideal structure and with double-cavity structure. The parameters are taken as the defect with the width t = 4.6 Å for ZPNRS, 3.3 Å for APNRS, 2.9 Å for ZGNRS, and 2.5 Å for AGNRS, and the length d = 3.3 Å for ZPNRS, 4.6 Å for APNRS, 2.5 Å for ZGNRS, and 2.9 Å for AGNRS. Here, the lengths between the defect region and the two lateral sides of main quantum wire Wa = Wb = 9.2 Å for ZPNRS, 8.3 Å for APNRS, 8.7 Å for ZGNRS, and 6.3 Å for AGNRS. The top-left inset describes the ratio k_G_/k_P_ as a function of temperature relative to the same chains across the ribbon.

Figure 3 shows the total thermal conductance σ divided by temperature *T* reduced by the zero-temperature universal value π^2^k_B_^2^/3*h* (*h* is Planck’s constant) as a function of temperature for different materials at low temperatures. The top-left inset describes the ratio *k*_G_/*k*_P_ as a function of temperature. Here, *K*_G_ and *K*_P_ are the thermal conductivities of GNRS and PNRS, and thermal conductivity *K* = σ*L*/(*DW*). *L* is the length of the nanoribbon, which is the same for GNRS and PNRS in this paper. *W* and *D* are the width and thickness of the nanoribbons, respectively. We can clearly see when stress-free boundary conditions are applied for the SH modes, ballistic transport for the 0 acoustic mode is possible. A quantized thermal-conductance plateau appears in a perfect quantum wire at very low temperatures, which arises from the 0 acoustic mode. With the increasing temperature, more acoustic modes with cutoff frequencies greater than 0 are excited and also contribute to the thermal conductance. The reduced thermal conductance increases monotonously, which qualitatively agrees with the experimental and theoretical results^33^,^39^. Note that the quantized thermal conductance plateau of GNRS is wider than that of PNRS. This can be mainly attributed to the higher cutoff frequency of mode 1 in GNRS. As a result of the higher cutoff frequency of mode 1 in GNRS, the higher temperature is needed to excite this mode, hence the wider quantized thermal-conductance plateau in GNRS. When temperature *T* increases, the total reduced thermal conductances are increased monotonically both in GNRS and PNRS. However, it is clearly seen from Fig. 3 that the total reduced thermal conductance of PNRS increases quicker than that of GNRS. This is because of the lower cutoff frequencies of acoustic modes in PNRS, more acoustic modes are excited in PNRS with increasing temperature. As a result, the reduced thermal conductance of PNRS is bigger than that of GNRS. Moreover, as more acoustic modes are excited, these acoustic modes with high energies in PNRS are scattered easier by the double-cavity scattering structures. Hence, the total reduced thermal conductance in PNRS with double-cavity structure is much less than that in PNRS without the double-cavity structure. It is interesting to note that the thermal-conductivity ratio *k*_G_/*k*_P_ is greater than 1 when the temperature T → 0 K, because *k*_G_/*k*_P_ = (σ_G_/σ_P_)(*D*_P_*W*_P_/*D*_G_*W*_G_) with the same *L* and chains across the ribbon width. At such low temperatures, only mode 0 is excited and thermal conductance σ_G_ = σ_P_ = π^2^k_B_^2^/3*h*. The ratio *D*_P_*W*_P_/*D*_G_*W*_G_ = 1.772 > 1 along the zigzag nanoribbon direction for *k* = 10 and *D*_P_*W*_P_/*D*_G_*W*_G_ = 2.0553 > 1 along the armchair nanoribbon direction for *k* = 7. This shows for the single acoustic mode, which transports the same thermal conductance in the nanoribbon with the same chains across the ribbon, the *k*_G_ in GNRS is bigger than *k*_P_ in PNRS. The ratio *k*_G_/*k*_P_ decreases monotonously with temperature *T* in the low temperature range. This is because with increasing temperature *T*, more acoustic modes with lower cutoff frequencies are excited in PNRS than in GNRS. Hence, the faster increase of the thermal conductance value in PNRS induces the monotonous decrease of the ratio *k*_G_/*k*_P_. However, it is clear from the top-left insets in Fig. 3 that the ratio *k*_G_/*k*_P_ in nanoribbons with double cavities is bigger than that in ideal nanoribbons. This can be understood from the transmission curves in Fig. 2. As more acoustic modes with higher energies are excited in PNRS, these acoustic modes are scattered more easily by the double cavities. Therefore, relative to the total transmission rates in perfect quantum structures, the total transmission rates decrease more obviously in PNRS than in GNRS, which restrains the fast increase of the thermal conductance in PNRS and leads to the slower decrease of the thermal-conductivity ratio *k*_G_/*k*_P_ in nanoribbons with double cavities.

**Figure 4:**
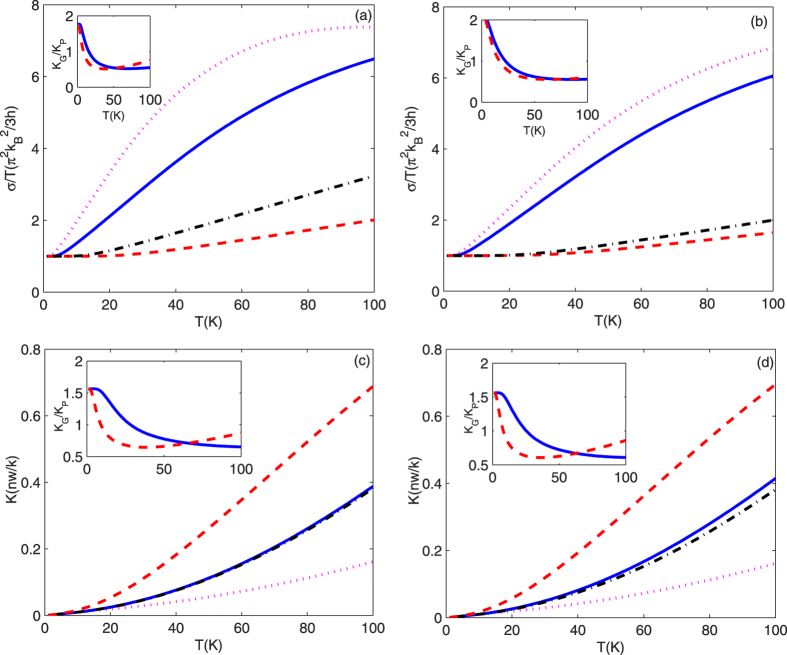
(**a,b**) correspond to the total thermal conductance divided by temperature K/T reduced by the zero-temperature universal value π^2^k_B_^2^/3h as a function of temperature along the zigzag direction and along the armchair direction. Figure 4(c,d) correspond to the total thermal conductance as a function of temperature along the zigzag direction and along the armchair direction, respectively. Solid and dotted curves of (**a**) correspond to the width W = 2.30 nm and 4.14 nm for ZPNRS, and dashed and dash-dotted curves of (**a**) correspond to the width W = 1.16 nm and 2.03 nm for ZGNRS, respectively. Solid and dotted curves of (**b**) correspond to the width W = 1.98 nm and 2.64 nm for APNRS, and dashed and dash-dotted curves of (**b**) correspond to the width W = 1.51 nm and 2.01 nm for AGNRS, respectively. Solid and dotted curves of (**c,d**) correspond to the width W = 1.60 nm for PNRS and GNRS, and the dashed and dash-dotted curves of (**c,d**) correspond to the width W = 5.00 nm for PNRS and GNRS. The top-left insets describe the ratio k_G_/k_P_ as a function of temperature relative to the same chains across the ribbon for (**a,b**) and the same width for (**c,d**).

In Fig. 4, we investigate the thermal conductance as a function of temperature with different ribbon widths. It is clear that when the transversal width becomes bigger, the length of quantum thermal-conductance plateaus is shorter and the reduced thermal conductance increases quicker with temperature. This is attributed to the fact that the longer transversal width can cause lower cutoff frequencies of the acoustic modes, and results in these modes being excited easier. These modes begin to contribute to the thermal conductance at such low temperatures. So the plateaus become shorter. In order to validate our calculations in the current method, the thermal conductance in ZGNRS with width = 1.6 nm (which equates 8-ZGNR-chain width) is calculated in Fig. 4(c). The result in the current method is consistent qualitatively with the result^23^ using the Green’s function method. Both the methods show the similar thermal conductance property in ZGNR at low temperatures despite the excited theory of the discrete phonon modes in quantum structure being not the same^23^,^29^. However, the thermal conductance in Green’s function method is bigger than that in current method with temperature T increasing. For example, the thermal conductance values are 0.01 nw/k, 0.02 nw/k, 0.03 nw/k, and 0.14 nw/k using current method, and are 0.017 nw/k, 0.04 nw/k, 0.08 nw/k, and 0.35 nw/k using Green’s function method^23^ when temperature T = 10.5 k, 20.5 k, 30.5 k, and 90.4 k, respectively. Even this, our calculations show that the thermal conductance values in ZPNR using current method are bigger than those in ZGNR using current and Green’s function methods when temperature T > 100 k. The total thermal conductances also both increase monotonously along the zigzag and armchair directions with the same widths owing to more acoustic modes being excited in the quantum structures. The thermal-conductivity ratio *k*_G_/*k*_P_ > 1 along both zigzag and armchair directions when temperature T → 0 K owing to only the low temperature quantum thermal conductance π^2^*k*_B_^2^/3*h* is transported in quantum structures and *k*_G_/*k*_P_ = *D*_P_*W*_P_/*D*_G_*W*_G_ > 1. The ratio *k*_G_/*k*_P_ decreases with lower temperature. This is because that ratio between the cutoff frequency of mode *n* in GNRS and mode *n* in PNRS is 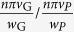, which equates to 3.443 in the zigzag nanoribbon and 3.767 in the armchair nanoribbon with the same width. This means that when the temperature reaches a certain temperature *T*_0_, the modes 1, 2, and 3 are excited in the PNRS, but the mode 1 is still not excited in the GNRS. So, the ratio *k*_G_/*k*_P_ = (σ_G_/σ_P_)(*D*_P_*W*_P_/*D*_G_*W*_G_) 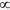 σ_G_/σ_P_ decreases at such low temperature. When the temperature is further increased, the modes with the cutoff frequencies greater than 0 are also excited in the GNRS. These modes start to contribute to the thermal conductance in the GNRS. The ratio *k*_G_/*k*_P_ increases with higher temperature.

**Figure 5:**
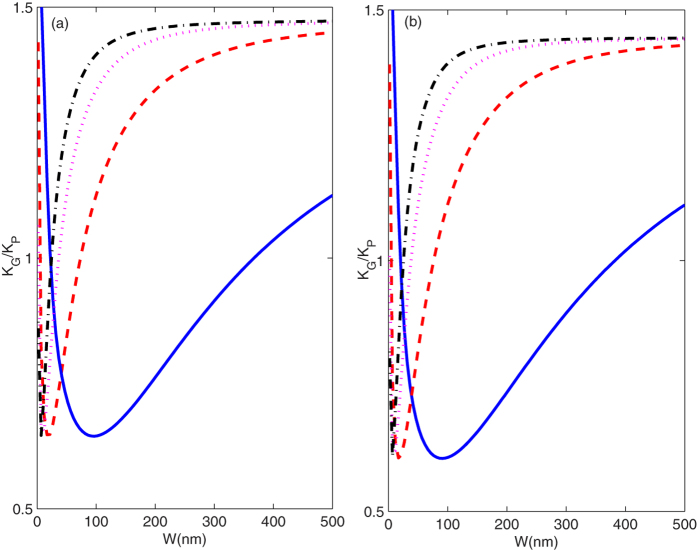
(**a,b**) correspond to the ratio k_G_/k_P_ as a function of width W along the zigzag and armchair directions. Solid, dashed, dotted, and dash-dotted curves correspond to the temperatures T = 2 K, 10 K, 20 K, and 30 K.

To compare the effect of the width on thermal conductivity in Fig. 5, we describe the ratio *k*_G_/*k*_P_ as a function of width *W* under different temperature *T*. Figure 5 shows that the ratio *k*_G_/*k*_P_ approaches 1.56 when temperature *T* = 2 K and width *W* → 0 nm. This is because at very low temperature and very narrow width, only the 0 mode is excited, the ratio *k*_G_/*k*_P_ = *D*_P_/*D*_G_ = 1.56 with the same *W* and length *L*. There are different threshold temperatures where the different modes with different cutoff frequencies 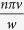 begin to be excited. With an increase in the width, the cutoff frequency 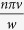 of mode *n* decreases. Because the ratio between the cutoff frequency of mode *n* in the GNRS and mode *n* in the PNRS is greater than 3, the modes with lower cutoff frequencies are excited with lower threshold temperature in the PNRS but the modes with higher cutoff frequencies are not excited in the GNRS. These excited acoustic modes start to contribute to the thermal conductance. Hence, in the very narrow width range, the ratio *k*_G_/*k*_P_ decreases with increasing width *W*. When width *W* is further increased, the modes with higher cutoff frequencies are also excited in the GNRS and begin to contribute to thermal conductance, the ratio *k*_G_/*k*_P_ increases with width *W*. When width *W* extends to the bulk limit, the quantum restriction influence on the thermal transport can be ignored. Hence, the ratio *k*_G_/*k*_P_ approaches a constant 1.56.

## Conclusion

The thermal transport properties in GNRS are systematically investigated using the continuum model of elastic waves at low temperatures. As a comparison, the thermal transport properties of PNRS are also provided. We observe that the transmission coefficient in PNRS is obviously larger than that in GNRS owing to the lower cutoff frequencies of acoustic modes in PNRS. Thermal conductance in PNRS is larger than that in GNRS containing the same carbon and phosphorene chains across the nanoribbon or with the same widths at low temperatures. However, the thermal conductivity of GNRS is larger than that of PNRS when the temperature → 0 K owing to the thin nature of GNRS. The ratio *k*_G_/*k*_P_ decreases with lower temperatures or for narrower nanoribbons, and increases with higher temperatures or for wider nanoribbons. The greater thermal conductance and thermal conductivity in PNRS originate from the lower cutoff frequencies of the acoustic modes. This is a promising result and provides information towards the potential for designing high-performance thermal phonon devices based on graphene and phosphorene.

## Additional Information

**How to cite this article**: Peng, X.-F. and Chen, K.-Q. Comparison on thermal transport properties of graphene and phosphorene nanoribbons. *Sci. Rep.* **5**, 16215; doi: 10.1038/srep16215 (2015).
